# Does the Humanitarian Sector Use Evidence-informed Standards? A Review of the 2011 Sphere Indicators for Wash, Food Security and Nutrition, and Health Action

**DOI:** 10.1371/currents.dis.40805a591152be1c1431b5dab43e516d

**Published:** 2018-10-30

**Authors:** Severine Frison, James Smith, Karl Blanchet

**Affiliations:** Health in Humanitarian Crises Centre, London School of Hygiene & Tropical Medicine (LSHTM), London, United Kingdom; Health in Humanitarian Crises Centre, London School of Hygiene & Tropical Medicine (LSHTM), London, United Kingdom; Health in Humanitarian Crises Centre, London School of Hygiene & Tropical Medicine (LSHTM), London, United Kingdom

## Abstract

Background: In 1997, the pursuit of greater accountability and effectiveness in humanitarian response prompted a multi-stakeholder collaboration to develop a set of indicators and standards to guide humanitarian practitioners, published later in the form of the Sphere Handbook. Twenty years after the first edition of the Handbook was developed, and in order to guide the 2018 revision, an assessment of the evidence base for current Water, Sanitation and Hygiene (WASH), Food Security and Nutrition, and Health Action indicators, as compared to evidence collated by the 2015 LSHTM Humanitarian Health Evidence Review (HHER), was conducted.

Methodology: In order to assess the utility of the Sphere indicators as a tool with which to monitor and evaluate humanitarian activities, indicators from the WASH, Food Security and Nutrition, and Health Action chapters of the Sphere Handbook were analysed and classified according to the SMART criteria. Each indicator was then assessed based on existing evidence related to the effectiveness of humanitarian health interventions as compiled in the HHER.

Results: Of the 159 Sphere indicators intended to guide humanitarian response, only 2 met all of the SMART criteria. The remaining 157 did not provide any time indication for the measurement of the indicator. Furthermore, only 11 standards (23%) and 14 indicators (8%) are supported in part by 33 studies identified in the HHER. Less than one third of studies captured by HHER that explore interventions related to WASH, nutrition, or health could be linked to existing Sphere indicators.

Conclusion: It is not possible to adequately link the 2011 Sphere indicators and standards to their sources in their current constitution, and they are not sufficiently evidence-informed. In the absence of clear measurement definitions, they do not provide necessarily detailed guidance. While recognising that a number of indicators have emerged as a combination of empirical evidence, expert experience, and “common sense”, a focus on fewer indicators, each better defined, is likely to enhance the practical application of the Sphere Handbook in humanitarian settings.

## Introduction

**Evidence-Informed Humanitarian Response & The Sphere Standards**

The pursuit of effectiveness and ethical practice in humanitarian health response, coupled with demands for improved accountability, have prompted calls to improve the evidence base for humanitarian interventions 1^,^^2^. In turn, greater attention has been paid to the existing evidence base related to the effectiveness of humanitarian interventions in crisis-affected contexts, with recent studies suggesting that a limited quality and quantity of evidence exists to support such interventions 3.

Evidence-informed decision-making in humanitarian crises is now recognised as a priority by humanitarian practitioners 4, and by leading organisations and policy-making institutions, with high-level commitments made by a number of the latter at the 2016 World Humanitarian Summit in Istanbul 5. The pursuit of evidence to support effective humanitarian interventions is not a new phenomenon, and is frequently traced back to the 1990s during which time a noticeable shift occurred from a general acceptance of humanitarian activities as inherently “good”, to greater critique of humanitarian programmes perceived to be ineffectual and inefficient 6. The pursuit of greater accountability and effectiveness in humanitarian response during this period prompted a multi-stakeholder collaboration that would lead to the launch of the Sphere Project in 1997.

The Sphere Project is responsible for the development and periodical update of the Sphere Handbook, which comprises both a Humanitarian Charter and Minimum Standards in Humanitarian Response 7. The first edition of the Sphere handbook (first trial edition in 1998; first final edition in 2000), with the development of best technical standards in particular, was seen as valuable and necessary but engendered criticism. The two main criticisms were that: i) although it had sought to promote a human rights-based approach, it failed to link the minimum standards to human rights principles, with the emphasis placed on technical standards at the expense of other humanitarian concerns such as accountability and protection 8^,^^9^^,^^10^^,^^11^; and ii) “one size does not fit all”, with many contexts requiring an adaptive and creative response 8^,^^10^^,^^12^^,^^13^. Later editions (2004 and 2011) attempted to tackle these issues with debatable success 9^,^^14^. Despite these critiques, twenty years since the formation of the Sphere Project, the Sphere Handbook is now widely recognised for its common principles and universal minimum standards for humanitarian response 15.

At its inception, the Sphere Project identified a set of minimum standards related to four key lifesaving sectors: water supply, sanitation and hygiene promotion (WASH); food security and nutrition; shelter, settlement and non-food items; and health action. The minimum standards have been described as ‘evidence-based’ and are intended to reflect ‘sector-wide consensus on best practice in humanitarian response, derived from the principle that all crisis-affected people have a right to a dignified life’ 16. The standards are qualitative in formulation, and specify a minimum level of response that should be achieved in the delivery of humanitarian assistance. Each standard is supported by one or more key indicators, along with key actions and guidance notes, which are intended to act as a measure of progress towards attainment of the associated standard.

Prior to the fourth revision of the Sphere Handbook, a survey commissioned by Enhancing Learning and Research for Humanitarian Assistance (ELRHA) and conducted in collaboration with the Sphere Project was conducted to assess knowledge of the Handbook and the extent of its contemporary use, along with views on its structure and content 17. This survey found that the Handbook remains a useful resource for humanitarian practitioners, and that there remains interest in its improvement. To further inform the revision of the technical standards of the Handbook, and to explore the extent to which one of the humanitarian sectors’ most widely disseminated and well-known resources is evidence-informed, this study assessed the evidence base that supports the current Sphere standards and indicators. As such, two objectives were proposed: 1) to explore both the utility of the Sphere indicators as a tool for the assessment of humanitarian activities; and 2) to examine the evidence-base supporting the Sphere standards and indicators for WASH, Food Security and Nutrition, and Health Action, following comparison with evidence generated by the LSHTM Humanitarian Health Evidence Review (HHER).

## Methodology

In order to assess the utility of the Sphere indicators as a tool with which to monitor and evaluate humanitarian activities, indicators from the WASH, Food Security and Nutrition, and Health Action (excluding Health Systems) chapters of the Sphere Handbook were analysed and classified according to the SMART criteria (see **Table 1**). These three chapters were chosen as they are closely comparable with evidence synthesised in the HHER. Each indicator was classified as follows: SMART criteria met (green); SMART criteria met with supporting guidance notes and / or appendices (yellow); not SMART (red). For the purpose of this analysis, the “R” component was interpreted either as relevant, or realistic. This expanded definition was adopted as a number of the Sphere indicators may be unrealistic, or only possible to measure in very specific settings such as in small, well organised refugee camps. While it is clear that humanitarian assistance is intended to reach all crisis-affected people, it is rarely realistic to fully achieve indicators that use language such as “all users”, “all staff”, “all disaster-affected people”, “all targeted beneficiaries”, “at all times”, and “no cases of health hazards”.

**Figure:**
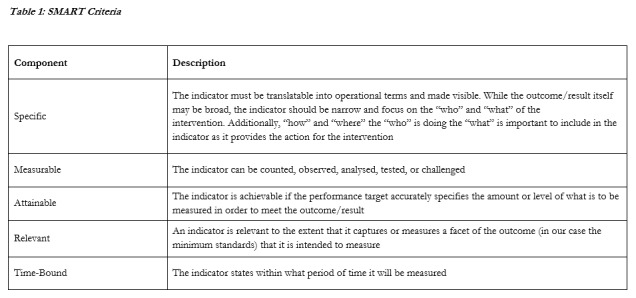
**Table 1:** SMART Criteria 18^,^^19^

Following classification of each of the indicators, evidence for humanitarian health interventions as collated in the 2015 HHER (capturing research published between 1980 and 2015) 3^,^^20^, was examined and matched to the relevant Sphere standard or associated indicator from the WASH, Food Security and Nutrition, and Health Action chapters. A detailed overview of the HHER, including the methodology, search strategy, key findings, and limitations, has been published elsewhere 3^,^^20^. In summary, the HHER comprised a series of systematic literature reviews of the evidence base for health interventions in humanitarian crises in low and middle income countries for the following health topics: communicable disease control; WASH 21; nutrition; sexual and reproductive health (SRH), including gender-based violence (GBV) 22; mental health and psychosocial support; injury and physical rehabilitation 23; non-communicable disease (NCD) 24; health services; and health systems.

Once matched, the following information was provided for each study that provided evidence to support a Sphere standard or indicator: author, year of publication, study design, study category, and a basic summary of key findings. Study categories were defined as category A if the study reported a statistical association between an intervention and health-related outcomes, and category B if the study measured changes in health-related outcomes, but did not report a statistical association.

## Results

***Classification of the Sphere Indicators***

The WASH, Food Security and Nutrition, and Health Action (excluding health systems) chapters of the Sphere Handbook report a total of 48 standards and 159 associated indicators, comprising 13 minimum standards and 58 indicators for WASH, 18 minimum standards and 63 indicators for Food Security and Nutrition, and 17 minimum standards and 38 indicators for Health Action.

Only two indicators (both for Health Action) of the 159 indicators report a loosely time-bound element: that assessment should take place following completion of a measles campaign for the measurement of measles and vitamin A coverage; and once routine Expanded Programme of Vaccination (EPI) services have been re-established for Diphtheria, Pertussis and Tetanus (DPT) coverage. For ease of further analysis, “Time” was dropped from the SMART criteria, such that all of the remaining indicators was assessed against their Specific, Measurable, Attainable, and Relevant (SMAR) qualities.

There is a notable discrepancy between chapters in the way that indicators are constructed. More than half of the WASH (51.7%; 30 of 58) and the food security and nutrition (57.1%; 36 of 63) indicators can be categorised as “SMAR”, or “SMAR with guidance notes and/or appendices” with a larger proportion of “SMAR with guidance notes and/or appendices” for the food security and nutrition compared to the WASH chapter (19.0% versus 8.6%). In contrast, the health action chapter is supported by a much larger proportion of SMAR or SMAR with guidance notes and/or appendices indicators (81.6%; 31 of 38) (See **Fig. 1**).

**Classification of the 159 Sphere indicators by chapter:**
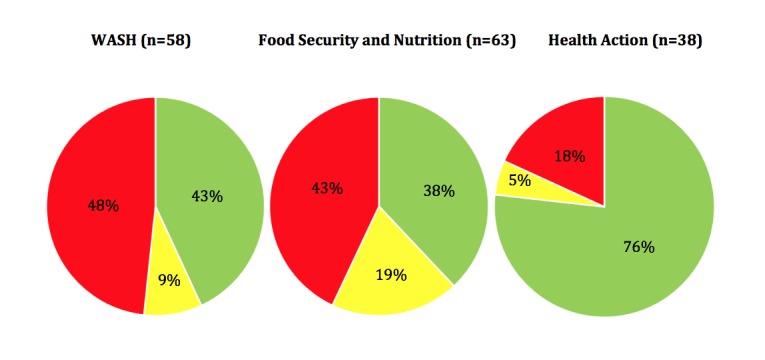
Green=SMAR; Yellow=SMAR with guidance notes and/or appendices; Red=Not SMAR

***Assessment of Supporting Evidence from the Humanitarian Health Evidence Review***

The HHER included 6 studies related to WASH, 77 studies related to Nutrition, and 236 studies related to Health (151 studies on communicable diseases, 8 on non-communicable diseases, 15 on sexual and reproductive health, and 62 studies on mental health). Very few of these studies provide evidence for the standards and indicators listed in the Sphere Handbook.

The WASH chapter of the Sphere Handbook contains 13 minimum standards and 58 indicators. Of the 6 studies included in the HHER, 4 studies support 3 (5.2%) of the indicators included in the Sphere Handbook and 3 (23.1%) of the standards. Four studies support directly 3 of the indicators associated with two of the water supply standards, and one of the hygiene promotion standards (see **Table 2**). All 4 studies measured statistical associations between an intervention and diarrhoea (i.e. category A). While the included studies provide some evidence, they do not support fully the associated indicator. For example, a study on soap distribution and diarrhoeal disease does not include all “hygiene items”, while the study on bucket provision and diarrhoeal disease does not specify a size or number of buckets.

**Figure:**
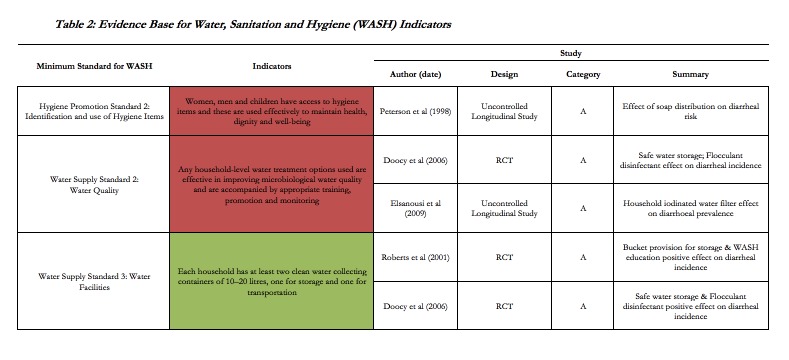
**Table 2:** Evidence Base for Water, Sanitation and Hygiene (WASH) Indicators

The Food Security and Nutrition chapter of the Sphere Handbook contains 18 minimum standards and 63 indicators. Of the 77 studies included in the review, 8 provide evidence for 7 (11.1%) of the 63 indicators, and 5 (27.8%) of the standards (see **Table 3**). While it should be noted that food security was not included in the HHER, 6 of the included studies support 5 food security indicators. All of the studies provide evidence in direct support of the indicators and 7 measure a statistical association between intervention and health outcome (acute malnutrition, micronutrient deficiencies, underweight, stunting), while the remaining paper measured a change in the prevalence of acute malnutrition but did not report a statistical association (category B). As with the evidence in support of the WASH indicators, the evidence only partially supports the selected Food Security and Nutrition indicators.

**Figure:**
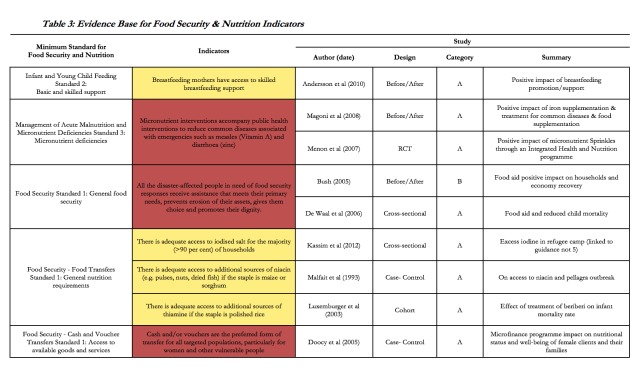
**Table 3:** Evidence Base for Food Security & Nutrition Indicators

The Health Action chapter contains 17 minimum standards and 38 indicators (excluding Health Systems). Of the 236 studies included in the review, 21 support to some extent 4 (10.5%) of the 38 indicators (see **Table 4**). Two indicators related to measles and routine EPI vaccination are supported by some evidence related to the positive impact of the vaccination itself, but with no supporting evidence regarding the coverage that should be achieved. Of the 21 supporting studies, 12 measure statistical associations between interventions and health outcomes (category A), while 9 measure changes in health outcomes, but do not report statistical associations (category B).

**Figure:**
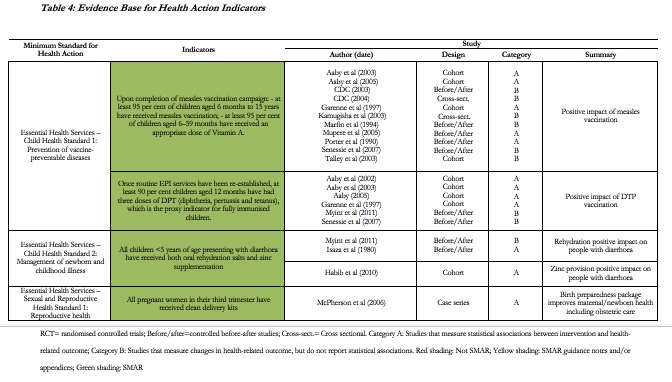
**Table 4:** Evidence Base for Health Action Indicators

## Discussion

Only 2 of the 159 indicators of the WASH, Food Security and Nutrition, and Health Action (excluding health systems) chapters of the Sphere Handbook were assessed as being SMART. The 157 remaining indicators did not provide any time indication for the measurement of the indicator. Excluding time bound information, 97 of 159 indicators (61%) were defined as SMAR and thus can be used as complete measurement tools. According to the Sphere Project, indicators “are ‘signals’ that assist in determining whether a standard has been attained. They provide a way of measuring and communicating the processes and results of key actions”. As such, the way in which many of the current Sphere indicators are phrased represents a weakness in terms of their operational applicability as tools for effective monitoring and evaluation. Furthermore, discrepancies were noted between chapters: a larger proportion of SMAR indicators were found in the Health Action chapter compared to WASH and the Food Security and Nutrition chapters (82% versus 52% and 57% respectively). While described as ‘evidence-based’ 16, only 11 standards (23%) and 14 indicators (8%) are supported in part by 33 studies identified in the HHER. Less than one third of studies captured by HHER that explore interventions related to WASH, nutrition, or health were linked to existing Sphere standards and indicators.

These findings are broadly in keeping with other studies that have sought to both qualify and quantify the evidence base that informs humanitarian interventions 3^,^^18^. A 2009 report on priority indicators in complex emergencies included an assessment of the Sphere indicators (based on the 2004 edition) and reported that of 346 indicators, 224 (65%) were not quantifiable, 48 (14%) were quantifiable but could not be supported by a search of the published literature, while 55 (13%) were supported by data 18. In 2017, Blanchet et al. 3, reported that the quantity and quality of evidence for various health interventions in humanitarian crises remains inadequate.

The use of indicators is essential in assessing the impact of an intervention. The Sphere Project tend to focus on process indicators (such as drug doses supplied, clinics supported or staff trained) or outcome indicators (such as clinic attendance) rather than impact indicators (morbidity/mortality reduction), which may raise fewer issues, as many such interventions have a vast literature detailing positive impact (for example measles vaccination or Vitamin A supplementation) 25.

The lack of evidence to support certain humanitarian interventions is largely attributable to challenges associated with the conduct of research in insecure settings, but also to the use of inappropriate methodologies and unsuitable study designs by many researchers attempting to investigate the effectiveness of humanitarian interventions 26. Various solutions, including the introduction of innovative methodologies and tools, may assist in the development of an improved evidence base for humanitarian assistance 27.

In the absence of a strong empirical evidence base, the authors of this paper recommend a thorough process of consultations with experts, with a detailed description of the consultation process and rationale for decisions. Accordingly, the Sphere Project suggests that “evidence-based” includes not only scientific evidence but also expert opinion and sectoral consensus. Although we agree that expert opinion and consensus may establish the foundation for some indicators, we have not adopted the same definition for the purpose of this analysis 28. We believe that greater transparency of the basis for, and origin of, the standards and indicators, and their classification based on their empirical, expert consensus, or “common sense” origins would be beneficial. Furthermore, if the classification of indicators were to be presented in a non-numerically ordered manner (i.e. not as 1, 2, 3 etc. as is currently the case), we would minimise the risk of implying a rank hierarchy of different types of evidence.

The authors of this paper have some concerns related to the number of indicators (159 in total for WASH, Food Security and Nutrition, and Health Action) in the current Sphere Handbook. In 2011, the US Centers for Disease Control (CDC) developed the Measurement of Selected Sphere Indicators (MeSSI) project for humanitarian response 19. Drawing from expert consensus they selected 50 priority Sphere indicators and proposed a definition for each, including a numerator and denominator, along with measurement methods. Unfortunately, this project was discontinued. We recommend that future iterations of the Sphere Handbook should attempt to reduce disparities between the different chapters, and prioritise the most important indicators (core SMART indicators), while providing a compendium of indicators that provides clear guidance on how each are to be measured and calculated. While it has been suggested that the presence of non-measurable indicators in the Sphere Handbook ensures that the issues they address are not forgotten, we would suggest that such “indicators” be reclassified as “principles”.

Nevertheless, it is important to note that since its launch in 1997 the Sphere Project has had a tremendous influence on humanitarian practice by defining minimum standards and situating accountability as a core principle in humanitarian practice. As demonstrated by a survey conducted by the authors of this paper and published in a companion report 17, there is no doubt that the Sphere Project is highly valued and needed by the humanitarian community. Over 70% of the 355 humanitarian professionals who participated in the survey strongly agreed or agreed that the Sphere Handbook: is a useful tool for the monitoring of projects; is a convenient source of information; is a good education tool; is likely to improve the quality of interventions; is a key tool for humanitarian beginners; and acts as a concrete translation of the humanitarian principles into practice. This strong consensus on the utility of the Sphere Handbook lends weight to the need for a clearer and more comprehensive evidence-informed approach.

Following an earlier evaluation of the Sphere Project in 2004, Van Dyke and Waldman expressed a need to revise the standards, clarify the difference between standards and indicators, and a need to rely more heavily on evidence 29. The findings of this study further explore the (in-)appropriateness of the current Sphere standards and indicators, and emphasise the need for an in-depth revision of the current Sphere handbook. In 2018, a revised version of the Sphere Handbook is likely to be published. To ensure that the new version of the handbook takes into account the results of this study, several meetings were organised with members of the Sphere Project. It is expected that the revised 2018 version of the Handbook will review existing evidence for the standards and indicators, and that indicator development will be documented following consultation with experts, with the SMART criteria as a guiding framework. Further operational research is required following the publication of the 2018 Sphere handbook to better understand the usability and relevance of the newly selected set of indicators.

Following the publication of the latest Sphere Handbook in 2011, the humanitarian community has acknowledged a series of new challenges. The number, intensity, and scale of humanitarian emergencies has changed in the last ten years 30. A growing appreciation of a long-established shift towards urbanisation and the challenges such a trend poses to humanitarian response, along with the changing needs of crisis-affected populations, particularly in relation to greater recognition of non-communicable diseases 31, have rendered many existing humanitarian standards and approaches redundant. It is therefore important that the new version of the Sphere Handbook recognises emerging epidemiological patterns and social phenomena (e.g. urban displacement, populations in transit, crises in middle-income countries) in order to remain relevant as a tool to guide humanitarian response.

Finally, it is important to note that the HHER only gathered published, quantitative evidence from crisis-affected settings. As such, not only did the HHER not recognise evidence that has emerged from expert consensus or “common sense” approaches, but it also did not capture evidence from stable settings, some of which has been applied in the development of the current Sphere standards and indicators.

## Conclusion

The Sphere Handbook was conceived in 1997 by a group of non-governmental organisations, in part to respond to the overall lack of accountability experienced during, and in the aftermath of, the Rwandan genocide in 1994. The Sphere Project developed from a recognition amongst humanitarian actors of the need for greater accountability and effectiveness in humanitarian response. Twenty years later, these concepts that underpin the Sphere Handbook remain as relevant as ever, in a time when humanitarian actors require up-to-date guidance to assist in their response to established and emerging issues, such as the management of non-communicable diseases in urban, middle-income contexts.

This study has demonstrated that humanitarian initiatives such as the Sphere Project must make better use of existing evidence, and document all decisions made by experts, during the development and revision of humanitarian standards and indicators. The Sphere standards and indicators published in 2011 were not sufficiently robust nor adequately evidence-informed, and do not stand as incontestable guidance, in light of the lack of clear measurement definitions that leave room for interpretation. We hope that our recommendations for greater transparency in the basis for, and origin of, the humanitarian standards and indicators, and a more refined selection of core SMART-compliant indicators, will be addressed in the next edition of the Sphere Handbook, due to be published in 2018. These recommendations, and outputs from the many expert consultations held worldwide, have the potential to reaffirm the relevance of the Sphere Handbook as one of the leading sources of technical guidance for humanitarian response.

## Corresponding Author

Karl Blanchet: karl.blanchet@lshtm.ac.uk

## Competing Interests

The authors declare no competing interests. The study funding source had no role in the analysis of the data, or the publication of results.

## Data Availability

All data used in this review is cited in-text.

## Appendix

**Figure:**
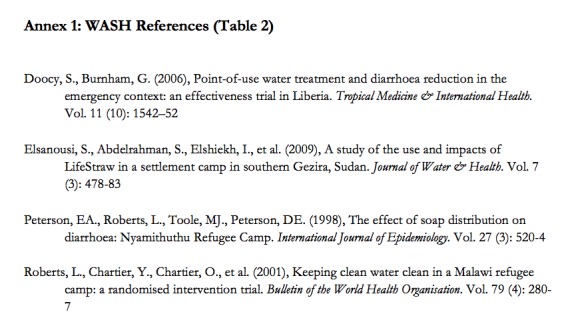
**Appendix I:** WASH References (Table 2)

**Figure:**
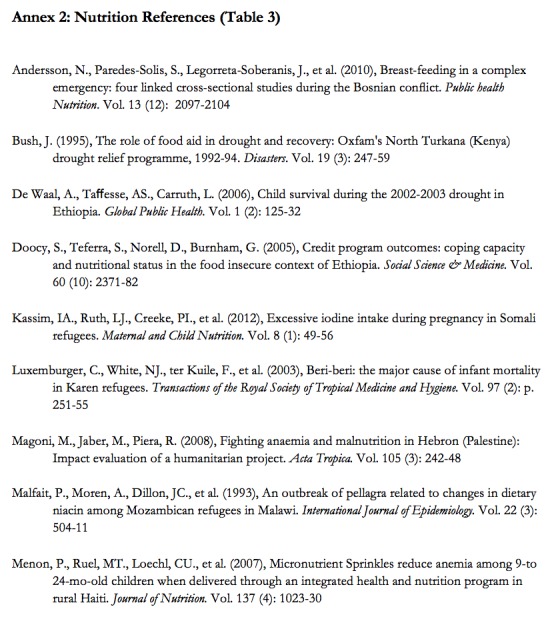
**Appendix II:** Nutrition References (Table 3)

**Figure:**
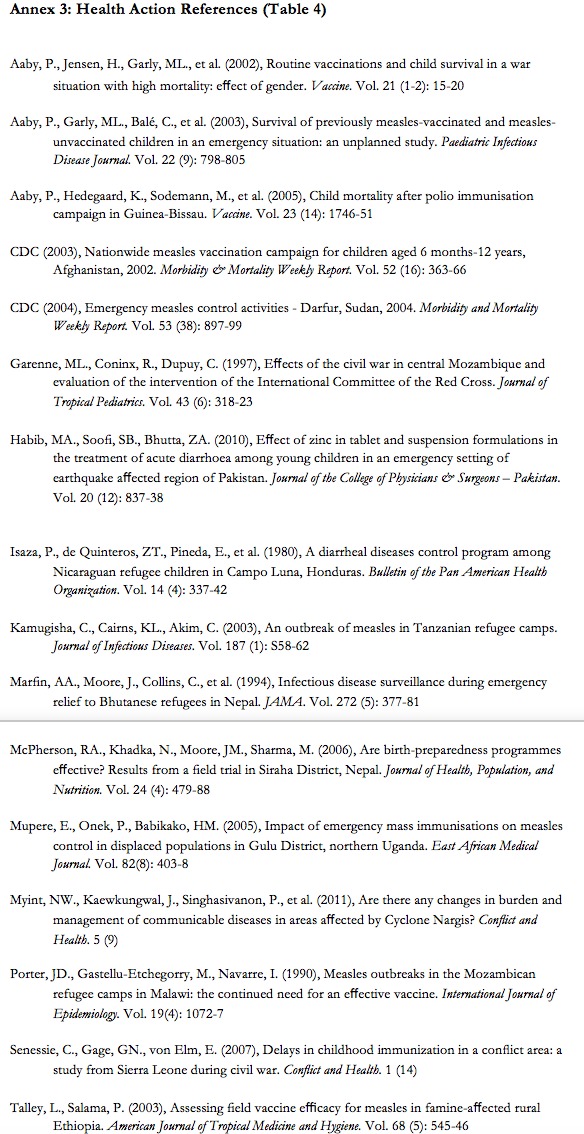
**Appendix III:** Health Action References (Table 4)
